# Surgical Approaches Based on Biological Objectives: GTR versus GBR Techniques

**DOI:** 10.1155/2013/521547

**Published:** 2013-06-13

**Authors:** Gaia Pellegrini, Giorgio Pagni, Giulio Rasperini

**Affiliations:** ^1^Department of Biomedical, Surgical and Dental Sciences, University of Milan, Milan, Italy; ^2^Foundation IRCCS Ca' Granda Policlinic, Milan, Italy

## Abstract

Guided tissue regenerative (GTR) therapies are performed to regenerate the previously lost tooth supporting structure, thus maintaining the aesthetics and masticatory function of the available dentition. Alveolar ridge augmentation procedures (GBR) intend to regain the alveolar bone lost following tooth extraction and/or periodontal disease. Several biomaterials and surgical approaches have been proposed. In this paper we report biomaterials and surgical techniques used for periodontal and bone regenerative procedures. Particular attention will be adopted to highlight the biological basis for the different therapeutic approaches.

## 1. Introduction

Guided tissue regeneration (GTR) and guided bone regeneration (GBR) are surgical techniques performed to regenerate, respectively, the tooth supporting tissues (GTR) and the alveolar bone in edentulous areas (GBR). 

Aim of GTR is the treatment of teeth affected by periodontal disease that induced loss of periodontal tissues and formation of infrabony defects. This procedure aims at the reconstruction of a periodontal ligament (PDL) with well oriented and organized collagen fibers inserted in newly formed cementum and newly regenerated alveolar bone [1]. The goals of GBR are (i) maintenance of the postextractive alveolar ridge volume that spontaneously reduces following tooth extraction, (ii) the reconstruction of the alveolar bone, that was lost following tooth extraction with the intent of realizing an implant supported prosthetic reconstruction or improved the aesthetics of edentulous areas, (iii) the correction of peri-implant dehiscences or fenestrations, and (iv) the reconstruction of the peri-implant bone that was lost following peri-implant disease [2]. 

From a biological and clinical standpoint, GTR and GBR share several important prognostic factors including stabilization of the blood clot [3, 4], ability to achieve primary intention wound healing, isolation of the defect from the gingival soft tissues, and space provision [5, 6]. Recently GTR techniques have progressed toward the use of minimally invasive approaches optimizing wound closure, limiting patient morbidity, and reducing need for regenerative materials including membranes [7–9]. Moreover, both GTR and GBR benefit from advances in regenerative medicine including the use of growth factors [10], gene therapy, and cell therapy approaches [11, 12].

The aim of the present paper is to review biomaterials (i.e., membranes, growth factors) and surgical techniques used for periodontal and bone regenerative procedures, with particular attention to highlight the biological basis that constitute the rationale for the use of different therapeutic approaches.

## 2. The Concepts of Cell Occlusion and Space Provision

Nyman and coworkers introduced GTR in the clinical practice with an experiment [1] that originated from Melcher's hypothesis that only cells originating from the periodontal ligament and repopulating the root surface during healing could regenerate the tooth supporting structures [13]. The authors applied an occlusive membrane between the gingival connective tissue/epithelium and the PDL/alveolar bone to prevent the migration of dentogingival epithelium and gingival connective tissue cells into the defect along the curetted root surface. Progenitor cells originating from the adjacent PDL and alveolar bone were therefore enabled to colonize the blood clot and induce periodontal regeneration. After 3 months of healing the histological analysis revealed the formation of new attachment in coronal direction to the level of 5 mm coronal to the alveolar bone crest. Later on, Dahlin et al. [14] successfully applied the biological principle of compartmentalization, to bone regeneration introducing guided bone regeneration (GBR).

*Barriers in GTR/GBR.* Several nonresorbable and resorbable membranes were proposed to provide isolation of the defect against gingival soft tissue invasion. Nonresorbable membranes including titanium foils and expanded polytetrafluoroethylene (e-PTFE) with or without a titanium reinforcement were evaluated (for review please refer to [15]). These biomaterials are biocompatible, inert [16, 17] and do not elicit immunological reactions that may interfere with the regenerative process. The titanium frame, when adopted, generates a mechanical support for the soft tissues over the defect to be regenerated and prevents the collapse of the mucosa into the wound area.

Space provision plays a fundamental role in both periodontal and bone regeneration [18]. Studies demonstrated that the use of titanium reinforced membranes alone or with a filling material results in significant bone formation even in large nonspace-maintaining implant dehiscences [19, 20]. One of the disadvantages of nonresorbable membranes is the necessity of an additional surgery for them to be removed. Another drawback of the original e-PTFE membranes was related to the unfavorable outcomes achieved when membrane exposure occurred including infection and limited bone regeneration.

In order to eliminate the drawbacks of nonresorbable membranes, several types of biodegradable membranes were introduced. Originally, resorbable membranes were mainly realized of polyesters (i.e., Polyglycolic acid-PGA, polylactic acid-PLA) and tissue-derived collagens [15]. Polymeric resorbable membranes maintain their maximum stability for about 14 days and then gradually loose their structural and mechanical properties within 30 days [21]. Polymeric membranes also showed limited biocompatibility [22]. Collagen membranes are more biocompatible than polymeric membranes, but they showed poor mechanical properties compared to nonresorbable membranes [15]. Clinical and preclinical studies compared resorbable and nonresorbable membranes in terms of defect fill in bone regenerative procedures and systematic reviews evaluated their use in both GTR and GBR [23–27]. In cases where membranes were not exposed, defect fill was greater when using e-PTFE than resorbable membranes. At the time, these results were explained considering the following features of e-PTFE membranes: (i) better space provision effect, (ii) controlled time of the barrier function, (iii) absence of inflammatory resorption that negatively influences tissue regeneration, and (iv) better surgical protocols with e-PTFE membranes originating from a longer experience [28]. PretzL et al. [29] demonstrated the stability of periodontal tissues regenerated with resorbable and nonresorbable membranes at 10 years after treatment. Carpio et al. [30] (2000) claimed that both collagen and e-PTFE membranes are suitable treatment options for GBR applications, but membrane fixation is fundamental in achieving a successful outcome of the treatment. Merli et al. [31] (2010) demonstrated that vertical bone regeneration obtained both with resorbable membrane supported by osteosynthesis plates and nonresorbable titanium reinforced e-PTFE membrane can be successfully maintained up to 3 years after implant loading.

The clinician must consider that e-PTFE membranes previously evaluated are no longer available on the market. Several advances in resorbable membranes technology have been introduced including cross-linked collagen membranes with longer resorption time and better biomechanical properties when compared to noncross-linked membranes [26, 32]. The use of resorbable membranes is now sustained by a large evidence and increased experience levels given the widespread use of these products in recent years. However, in a human study, intrabony defects were treated with GTR and resorbable collagen membranes, and histological evaluations revealed the formation of long junctional epithelium above newly formed cementum and periodontal ligament [33]. Furthermore the study observed that filling material was mostly embedded in connective tissue, without any evidence of bone regeneration [33].

More recently a high density PTFE membrane is being more closely evaluated. Because of its smaller pore size compared to e-PTFE membranes, d-PTFE seems to better withstand exposure to bacteria from the oral cavity, reducing the drawbacks of membrane exposure even when using this nonresorbable barrier [34, 35].

## 3. The Concept of Blood Clot Stability

To date a large number of clinical trials demonstrated the success of periodontal and bone regenerative procedures using membranes. However, the importance of defect isolation by occlusion membranes on tissue regeneration was questioned in several articles. In GTR, the formation of a long junctional epithelium as a consequence of periodontal repair as opposed to regeneration has been suggested to be more closely related to wound failure rather than to failure of defect isolation *per se* [36, 37]. Several studies reported on the critical role of an uncomplicated adsorption, adhesion, and maturation of the fibrin clot at the tooth-mucogingival flap interface to achieve a new connective tissue attachment and to prevent the downgrowth of the junctional epithelium [36, 38]. 

Recently, gingival stem cells suitable for periodontal and bone regeneration were isolated from gingival connective tissue [39, 40], and different GBR papers have suggested a possible role of periosteal cells and gingival stem cells in wound healing [41–43]. According to these studies, an occlusive membrane may not be the best choice as compared to porous membranes. The advantages of titanium reinforced ePTFE membranes noted in clinical studies may therefore be related more to space provision, and blood clot stabilization effects, instead of cell occlusion. Resorbable membranes do not have this self-maintaining space characteristic and may be used alone only in contained defects. Noncontained defects treated with resorbable membranes may therefore benefit from the combined use of a grafting material acting as a scaffold [44] as we will discuss briefly. 

*Filling Materials*. The biological rationale on the use of filling materials for periodontal and bone regenerative procedures is mainly based on their scaffolding, space-maintenance and blood clot-stabilizing properties. Several filling materials have been proposed. Autologous bone has osteogenic, osteoinductive, and osteoconductive properties, making of it the material of choice for bone regenerative procedures for many decades [45]. Compared to xenografts, autologous bone seems to accelerate the healing process in maxillary ridge defects [46]. However, autologous bone collection requires access to an additional surgical site as a donor, and unfortunately, following implantation, a stronger remodeling process and volumetric reduction than anorganic bovine bone take place [47]. In order to prevent autologous bone resorption during wound healing the use of membranes have been evaluated [48, 49]. Gielkens et al. (2008) [48] observed that both resorbable and nonresorbable membrane do not prevent onlay bone graft remodelling resulting in graft resorption. The authors also stated that membranes are necessary to secure particulate bone containment but do not prevent bone resorption [48].

The resorption rate of bone substitutes instead is quite slow [50]. 

Several studies observed a partial remodeling of xenograft particles (Bio-Oss, Geistlich, Wolhusen, Switzerland) also several years after treatment [51–53].

This data could be explained by the reduced levels of proinflammatory cytokines and growth factors produced by osteoblasts exposed to Bio-Oss [54]. Furthermore human and preclinical histological studies reported that xenograft materials can elicit an immunological-inflammatory process that may delay the formation of new bone in the grafted areas [52, 55]. 

In the regeneration of tooth supporting structures, autologous grafts demonstrated high potential for periodontal growth [56], but the current tendency of reducing flap extension by using minimally invasive approaches limits the appeal of this filling material.

In bone regenerative procedures instead, the use of autografts is still common; however, the efficacy of bone substitutes has been thoroughly validated for GBR as well. Studies comparing autogenous bone deproteinized bovine bone mineral in vertical bone augmentation failed to demonstrate significant differences between grafts in terms of bone gain [57, 58]. Further studies assessed the efficacy of using bovine bone grafts in combination with autogenous bone to decrease the resorption rate of the graft, to increase the long-term stability of the regenerated tissue, and to reduce the morbidity associated with extraoral donor sites [59, 60]. In a Cochrane review, Esposito et al. (2009) stated that some bone substitutes could be a preferable alternative to autogenous grafts and that patients prefer a bone substitute block over a block of autogenous bone taken from the iliac crest [61].

## 4. Surgical Techniques Evolution

Wound stability and primary intention closure of surgical flaps are of primary importance for the prognosis of regenerative procedures [4]. The close adaptation of the flap to the root seems to accelerate the periodontal healing process [38]. Furthermore, lack of papilla's primary intention closure results in membrane exposure—one of the most frequent complications occurring in GTR/GBR techniques—impairing the process of tissue regeneration [24].

In order to get blood clot stability and to prevent membrane exposure, more attention was given to the management of soft tissues, so that more refined and minimally invasive surgical approaches were designed. In 1985, Takei et al. proposed a flap technique aimed at preserving the interdental papilla and allowing easy primary intention closure of the palatal and vestibular flaps [62]. This approach was revised by Cortellini et al. (1995, 1999) who proposed to cut the papilla on the vestibular side only, so that the coronally advanced vestibular flap was sutured with less tensions to the palatal flap [63, 64]. This surgical approach combined with the use of the microscope and microsurgical instruments demonstrated an increased percentage of primary intention closure that resulted in important clinical results in terms of clinical attachment level gain with minimal recession [65]. In order to reduce surgical trauma, to increase flap stability and to apply microsurgical concepts, surgical approaches were proposed to limit the mesiodistal flap extension and the coronal-apical flap reflection [66, 67]. Wachtel et al. (2003) compared clinical performances of microsurgical flap alone or in combination with EMD and reported that both treatment modalities obtained a high percentage of primary flap closure and maximum tissue preservation [66]. Furthermore the combination of microsurgical approach and EMD resulted in better results in terms of PPD reduction and CAL gain. In the last years, evolutions of these papilla preservation techniques were proposed [7, 68]. Blood clot stabilization is increased by elevating a flap only on the buccal or on the oral side according to the defect position. The corresponding oral or buccal portion of the interdental papilla is left undetached to allow easy and more stable flap repositioning. Furthermore the blood supply of the interdental area seems to be better preserved [68]. Despite the use of regenerative materials, the great importance of surgical flap management and of blood clot stabilization on tissue regeneration was demonstrated in clinical trials that reported improvements in clinical and radiographic outcomes when minimally invasive surgical approaches were used alone or combined with regenerative biomaterials including: hydroxyapatite + membrane, EMD, and PDGF [9, 69, 70]. These microinvasive approaches are indicated when the aesthetic appearance of the area is acceptable and there is no need for coronally advanced flap.

Recently we suggested a novel surgical technique for the treatment of challenging infrabony defects when gingival recession is an aesthetic concern. By creating a stable soft tissue wall this technique allows to compensate for the lack of bone support, reaching both biological and aesthetic clinical success. The soft tissue wall technique has been described elsewhere [71]. Briefly, a horizontal incision is carried out at the base of the interdental papillae extending one tooth mesially and distally from the infrabony defect. A full-thickness trapezoidal flap is then elevated, and the remaining facial gingiva is deepithelialized. The defect is degranulated, and the root surface is carefully scaled and planned. The buccal flap's periosteum is dissected, and the flap is mobilized and coronally advanced to a level more coronal to the CEJ. Sling sutures are used to stabilize the flap. The root surface is now conditioned with EDTA gel and rinsed with saline solution. An enamel matrix protein gel is applied into the defect, and a tension-free primary closure of the interdental papilla is achieved using a 7-0 nonresorbable ePTFE internal horizontal mattress suture. Finally, the vertical releasing incisions are closed with interrupted sutures. The proposed technique has been proved to achieve CAL gain and PD reduction while improving aesthetics of the area by modifying the gingival margin outline. The ability to stabilize the wound healing environment in challenging one wall bony defects allows to achieve and preserve the space for regeneration without using any space-maintaining biomaterials [71].

As the defect size in bone augmentation procedures is often much extended, minimally invasive surgical approaches are seldom adaptable to GBR reconstructive therapy, and more invasive techniques have to be applied. Soft tissue management is a key factor to obtain wound primary closure in GBR techniques as well. Proper flap management allows to avoid membrane exposure and infection which may impair the regenerative procedure, especially when nonresorbable membranes are used [72, 73]. In order to reach better flap release and closure and prevent these adverse events, several surgical approaches were proposed [74–76]. A novel technique for the coronally advancement of a lingual flap was recently published [77]. In 2006, Merli et al. proposed a different approach based on osteosynthesis microplates as space provision devices associated with resorbable membranes [78]. This technique avoids the use of risky nonresorbable membranes; however, up to 3 years after loading a significant reduction of peri-implant bone was assessed and no differences were found with the patients treated with nonresorbable titanium-reinforced membranes.

To date, it is unclear which vertical and horizontal bone augmentation procedure is preferable. GBR is still technically demanding and clinical results are unpredictable [61]. Furthermore limitations of regenerative biomaterials (i.e., membranes and grafts) and difficulties in surgical approaches still persist with both GTR and GBR. Clearly, tissue regeneration requires 3 factors: (i) cells to produce the tissue and supporting structures; (ii) growth factors to orchestrate cell activity; (iii) scaffolds to provide space and a structure for extracellular matrix deposition.

Tissue engineering strategies are under evaluation both for GTR as well as for GBR treatments. These novel tissue engineering therapies include the delivery of bioactive molecules (EMD/growth factors), new scaffolding techniques, the stimulation of the selective production of growth factors using gene therapy, and the delivery of expanded cellular constructs.

## 5. Bioactive Molecules Delivery: EMD

Amelogenins are the major proteinic component of a form of extracellular matrix proteins with high affinity for hydroxyapatite and dental root surface [79]. During odontogenesis and development of tooth attachment apparatus, these proteins adsorb on the root surface and induce the formation of acellular cementum [80]. In 1997, a purified acid extract of enamel matrix proteins (Emdogain, EMD; Institut Straumann, Basel, Switzerland) was used to treat a human experimental defect, and the formation of new acellular extrinsic fiber cementum was assessed [81]. A further human histologic sample of a tooth treated for gingival recession with connective tissue graft (CTG) + EMD demonstrated the formation of woven bone and connective tissue anchored in the new cementum [82]. *In vitro* studies assessed how enamel matrix derivative stimulates PDL fibroblast and osteoblast proliferation-differentiation [83, 84].

Following these encouraging results, several trials were designed to assess the efficacy of enamel matrix derivative (EMD) on reducing pocket probing depth of infrabony and furcation defects and in the treatment of gingival recessions [85–88]. A recent systematic review evaluated the benefits of additional use of EMD in periodontal regenerative procedures. The authors stated that the use of EMD in the treatment of infrabony defects is superior in terms of CAL gain as compared to open flap debridement, placebo or root conditioning with 24% EDTA, and as effective as resorbable membranes. In the treatment of gingival recessions, the coronally advanced flap technique (CAF) + EMD resulted more effective than CAF alone, but no differences were found between the CAF + EMD group and the CAF + CTG [89]. In the treatment of furcation defects, EMD gives more reduction in horizontal furcation defect depth than the use of a resorbable membrane. Long-term clinical study confirmed that the clinical improvements obtained with the use of EMD can be maintained over a period of 10–15 years [86]. 

Studies on osteopromotive effects of EMD [84, 90] suggested a more extended range of clinical applications of this product in dental practice than the only tooth supporting regenerative therapy including: the bone and peri-implant bone regeneration [91]. Preclinical studies evaluated the effects of GBR in combination with or without deproteinized bovine bone mineral (DBBM) and/or an enamel matrix derivative (EMD) on bone healing and regeneration [92, 93]. The authors stated that the use of EMD does not positively affect the amount of new bone formation and that the predictability of bone formation in critical-size defects depends mainly on the presence or absence of barrier membranes (GBR). The combined use with deproteinized bovine bone mineral and/or enamel matrix proteins did not significantly enhance the potential for complete healing provided by the GBR procedure. 

It should be highlighted that in the context of tissue regeneration, EMD's beneficial effects seems to be mostly on formation of periodontal ligament and cementum, while its impact on new bone regeneration seems to be limited.

## 6. Bioactive Molecules Delivery: Growth Factors

The use of growth factors in dental surgery dates back to the introduction of platelet rich plasma (PRP) [94, 95] and plasma rich in growth factors (PRGF) [96–98]. With such techniques the patient's blood was centrifuged in order to enhance the concentrations of platelet's growth factors by 2 to 8 times. More recently recombinant Growth Factors have been introduced including platelet-derived growth factor (PDGF-BB), transforming growth factor-beta 1 (TGF-*β*1), insulin-like growth factor-1 (IGF-1), vascular endothelial growth factor (VEGF), endothelial cell growth factor (ECGF), fibroblast growth factor-2 (FGF-2), and bone morphogenetic proteins (BMPs). By using recombinant GFs purified solutions with much higher concentrations of a single GF or combinations of GFs can be achieved (1000 + times).

Bone morphogenic proteins (BMPs) are able to induce the differentiation of the host stem cells into bone forming cells (osteoinduction) [99]. RhBMP-2 absorbed in a collagen sponge has been successfully evaluated for alveolar ridge preservation after tooth extraction in both the posterior segments [100, 101] as well as in more challenging defects [102]. RhBMP-2 and RhBMP-7 seem to have great potential for GBR applications, although rhBMP-12 may be more appropriate for GTR [103].

RhPDGF-BB has been accepted by the FDA for regeneration of bone and PDL elements in guided tissue regeneration procedures. Good results have been showed by using this growth factor both in GTR [104–107] as well as in GBR such as socket grafting [108], localized grafting procedures [109], maxillary sinus augmentation, and vertical ridge augmentation [110]. 

FGF-2 has also been extensively evaluated mostly in periodontal applications [111–114]. Fibroblast growth factor (FGF)-2 displays potent angiogenic activity and mitogenic ability on mesenchymal cells especially on PDL cells and decreases alkaline phosphatase activity. Supposedly exogenous FGF-2 may act differently on PDL cells and gingival epithelial cells *in vivo* in terms of proliferative response, blocking epithelial downgrowth and stimulating PDL cells growth [114].

Other studies evaluated the use in periodontal therapy of platelet-derived growth factor (PDGF) in combination with insulin-like growth factor (IGF)-I [115], bone morphogenetic protein (BMP)-2 [116, 117], transforming growth factor (TGF)-b [118], osteogenic protein (OP)-1 [119], and brain-derived neurotrophic factor (BDNF) [120].

The use of recombinant growth factors in GTR and GBR has shown interesting results especially if you consider that because of the inflammatory environment in surgical areas, their presence in the wound area is confined to the first few hours. Recombinant growth factors initiate a cascade of events which is probably the explanation of the good results obtained in clinical studies. Anyway tissue engineering efforts are directed toward the possibility of extending the duration of growth factors in the wound area. 

## 7. New Scaffolding Technologies

Scaffolding matrices serve as three-dimensional template structures to support and facilitate periodontal tissue and bone regeneration.

Scaffolds should be biocompatible and should be able to provide adequate mechanical stability of the defect. A high porosity and surface-to-volume ratio with a well-interconnected open pore structure is recommended in order to provide an environment where space is created and maintained to allow cellular and tissue in-growth. Moreover scaffolds should degrade once tissue is allowed adequate time to regenerate [121]. 

For periodontal purposes particulate grafts of autogenous, allogenic, xenogenic, and even synthetic origin have been adopted with variable outcomes. For GBR purposes both particulate and block grafts have been adopted. Both particulate and block scaffolds can be adjusted to fill the defect and provide support and space maintenance for regeneration. Unfortunately the pattern of their structure can only be adapted, but in relation to the tissue to be regenerated they function as amorphous structures. Also, while blocks may have the ability of withstanding pressure applied from flaps, muscles, or other external stimuli over their surface, particulate grafts do not and require therefore to be used either in a self-contained defect or in combination with other tenting devices.

Proper tissue regeneration is facilitated by an optimal cell-tissue directionality which can be achieved through the development of emerging scaffold technologies [122, 123]. Recently, computed tomography or magnetic resonance imaging data have been used to define the anatomic geometry of a defect and CAD-CAP technologies used to create an image-based three-dimensional printed scaffold that is custom fit to the defect prior to the time of surgical intervention. Furthermore, the architecture of the scaffold can be defined to design the heterogeneous internal structure in a way to create region-specific variations in porous microstructures and scaffold surface topography, thereby altering material and biologic properties in specific regions of the scaffold, such as modulus, permeability, and cell orientation [124]. Studies have shown not only that with this technology it is possible to accomplish a 96% + precise fit between the scaffold and the defect surface, but also that the fiber-guiding scaffold allowed for predictable regeneration of oriented PDL fiber architecture, a greater control of tissue infiltration, and better organization of ligament interface when compared to a random porous scaffold [125]. With the ability to establish a three-dimensionally customizable microscaffold and macroscaffold architecture it is possible to accomplish another step toward the creation of biomimetic scaffold surfaces [126]. With this scaffold technology we are able to address specific periodontal functional requirements, such as PDL fiber orientation and tissue integration of cell- and gene-based technologies [125, 126].

## 8. Scaffolds for Delivery of GFs, Cells, and Gene Therapy

Another interesting application of new scaffolding technologies resides in the ability of conducting a sustained release of antibacterial substances, bioactive factors, or cells in order to, respectively, reduce chances for bacterial contamination, induce stimuli for tissue formation, or provide cells to the defect directly implicated with tissue neogenesis.

Feng and coworkers developed a localized and temporally controlled delivery system to achieve high local bioactivity and low systemic side effects of antibiotics in the treatment of dental, periodontal, and bone infections. Doxycycline was incorporated into PLGA nanospheres and incorporated into prefabricated nanofibrous PLLA scaffolds with a well interconnected macroporous structure. Different formulations resulting in different release kinetics were evaluated. The investigators were able to extend the release of Doxycycline to longer than 6 weeks with the potential of inhibiting growth of common bacteria such as *S. aureus* and *E. coli* [127]. 

Sustained release of PDGF-BB from several days to months was achieved through the incorporation of microspheres in scaffolds. By stimulating human gingival fibroblast DNA synthesis *in vitro* the researchers were also able to prove the released protein to possess biological activity [128]. Also, rhBMP-7 was encapsulated into PLGA nanospheres demonstrating a temporally controlled release kinetics and inducing significant ectopic bone formation throughout the scaffold, whereas passive adsorption of rhBMP-7 into the scaffold resulted in failure of bone induction [129].

Different cell source can be successfully implemented to promote desired tissue formation when provided with adequate support of cell function [130, 131]. Tissue-engineered scaffolds can provide adhesion and anchorage for interacting transplanted stem cells [132, 133]. Also, stem cell niches might be mimicked in order to regulate proliferation, differentiation, and dispersal of daughter cells into the surrounding tissue to participate in regeneration or provide trophic factors over a large volume [134].

Synthetic polymers have been studied as a localized gene depot for gene therapy applications. Via these approaches therapeutic levels of encoded proteins that limit unwanted immune response and potential side effects can be promoted [135].

Other scaffolding materials being tested in the GTR and GBR tissue engineering field include HA [136, 137], *β*-tricalcium phosphate [138, 139], and Hydrogels derived from collagen chitosan, dextran, alginate, or fibrin [140–142].

Despite the progress of the tissue engineering field in periodontal and dental implant therapy the adoption of these systems remains mostly reserved to preclinical and clinical research applications.

## 9. Cell and Gene Therapy

The more recent advances in tissue engineering research is the use of gene and cell therapy. It must be understood that despite the fact that these strategies are separate in theory often they are combined together or with other tissue engineering technologies as 3D scaffold printing.

Cell therapy approaches provide an additional source of cells in the area of interest, with the intent to be used as grafted cells (which will integrate into the patient's body) or when not intended for integration, as a source of growth factors [143, 144]. Somatic and stem cells can both be used in cell-based therapy. Somatic cells can be harvested, cultured and implanted in order to stimulate the generation of the new tissue. The advantage of using stem cells resides in their self-renewal capability and potency [145]. Also, a novel cellular approach developed making use of both cell and gene therapies for the production of induced pluripotent stem (iPS) cells has been recently evaluated.

Somatic cells as fibroblast-like cells derived from the periodontal ligament have been used to promote periodontal regeneration [146]. A study showed that oral-derived periodontal cells are able to stimulate alveolar bone formation *in vivo* [147]. When seeded onto three-dimensional polylactic-coglycolic acid scaffolds, cloned tooth-lining cemento-blasts, periodontal ligament fibroblasts, and dental follicle cells exhibited mineral formation *in vitro* [148] and immortalized cementoblasts delivered to large periodontal defects via a PLGA polymer carrier contributed to complete bone bridging and PDL formation, while dental follicle cells inhibited bone formation [149].

In another study skin fibroblasts transduced by the BMP-7 gene promoted the regeneration of periodontal defects including new bone, functional PDL, and tooth root cementum [150].

An optimal example of stem cell therapy is the use of tissue repair cells (TRCs also known as bone repair cells—BRCs or Ixmyelocel-T), an autologous source of stem and progenitor cells derived from the patient's bone marrow and cultivated using automated bioreactors to concentrations not achievable through a simple bone marrow aspiration was evaluated in socket healing. This cell construct is able to produce significant concentrations of cytokines and maintains the cells' ability to differentiate toward both the mesenchymal and endothelial pathway and produce angiogenic factors [151]. TRC therapy has been shown to enhance formation of highly vascular mature bone as early as 6 weeks after implantation when compared to guided bone regeneration with no serious study-related adverse event reported, and lower degrees of alveolar ridge resorption were noted [152].

For further information on cell therapy applications in craniofacial regeneration please refer to our recent review [153].

Gene therapy is a technique making use of different types of carriers (i.e., plasmids, retroviruses, adenoviruses, and adenoassociated) to inoculate a gene into host cells or implanted cells. The gene transduces for the desired protein (as a growth factor or an anti-inflammatory cytokine), so that the desired effect can be stimulated. Through gene therapy, Dunn and coworkers were able to maintain a sustained transgene expression of a recombinant adenoviral vectors encoding either the BMP-7 for up to 10 days at the osteotomy sites with nearly undetectable levels by 35 days. A surgically created defect around dental implants and treated with Ad/BMP-7 resulted in enhancement of alveolar bone defect fill, coronal new bone formation, and new bone-to-implant contact when compared to controls (Ad/Luc) [154]. In another study using a similar model, bone repair was accelerated by the use of Ad-PDGF-B and rhPDGF-BB delivery compared with Ad-Luc, with the high dose of Ad-PDGF-B being more effective than the low dose [155].

Gene therapy was originally designed for the treatment of diseases or disorders requiring transfer of genetic materials to introduce, suppress, or manipulate specific genes [156]. The therapeutic concept modulation of the host response in order to achieve regeneration of periodontium is compelling especially because of the advantages that this strategy may offer. Gene therapy ensures greater sustainability when compared to the application of a single protein or compound and reduces challenges associated with *ex vivo* protein expression and purification (i.e., palmitoylation, glycosylation). Moreover a transient and controlled delivery method could mimic more closely the natural biologic healing response [121]. Finally, gene therapy can be associated with other tissue-engineering strategies, therefore offering strong potential in regenerating complex tissue structures as the periodontal ligament and other tooth/implant supporting structures.

## 10. Conclusions

Tissue and bone regenerations are performed by clinicians to reduce periodontal pocket depth, to achieve a more favorable crown/root ratio, and to have a long-term soft tissue stability and for a proper (from a prosthetic point of view) implant placement. The available treatment options to reconstruct the tooth supporting structures include: (i) resorbable/nonresorbable barrier membrane, (ii) grafts, (iii) delivery of bioactive molecules, and (iv) different flap design that promote flap stability. Anatomical features of the defect guide the clinician in the treatment choice. Narrow and space-maintaining defects are more suitable to be treated with EMD than wide and nonspace-maintaining defects requiring membranes and filling materials for space provision and wound stabilization. Periodontal regeneration performed following the techniques cited above is an affordable and predictable therapy [44, 86]. Periodontal regeneration also allows to preserve aesthetics and masticatory function and may avoid need for replacement with implants [44, 86]. Patient's features (nonsmoking, good oral hygiene, and maintenance) are crucial for tooth maintenance [86, 157].

In case of severe atrophic mandibles, several techniques were proposed including: (i) resorbable/nonresorbable barrier membrane, (ii) osteosynthesis microplates, (iii) grafts and bone blocks, (iv) growth factors (i.e., BMP-2), and (v) distraction osteogenesis. To date, it is unclear which is the best alternative for vertical and horizontal bone augmentation, complications of GBR are common, and the prognosis still largely depends on ability of the operator [61]. To overcome these disadvantages, placement of short implants (<10 mm of length) in dimensionally limited alveolar ridges may be the preferred option in the treatment of many clinical cases. The efficacy of this treatment modality has been confirmed at least in the short-term period [158]. 

Considering the data reported previously, tooth preservation is a preferable therapeutic option whenever possible. Advances in tissue engineering technologies are quickly changing the scenario of treatment possibilities both in periodontal and implant therapy suggesting the possibility of achieving optimal and predictable tissue regeneration even in cases that could not be treated with currently available technologies.
